# The impact of stress, anxiety and depression on stomatognathic system of physiotherapy and dentistry first‐year students

**DOI:** 10.1002/brb3.1797

**Published:** 2020-08-29

**Authors:** Joanna Elżbieta Owczarek, Katarzyna Małgorzata Lion, Małgorzata Radwan‐Oczko

**Affiliations:** ^1^ Department of Oral Pathology Wroclaw Medical University Wroclaw Poland; ^2^ Menzies Health Institute Queensland Griffith University Nathan QLD Australia

**Keywords:** anxiety, depression, stomatognathic system, stress, students

## Abstract

**Introduction:**

It is well proven that psychoemotional factors play causative role in development of many civilization diseases. Psychosocial stress is effecting with development of certain oral parafunctions like teeth grinding, bruxism, or cheeks biting. Eventually, all of those parafunctional activities may cause increase of masticatory muscles tone and provoke its intensification. Literature indicates that students must cope with greater level of stressful situations than the average representative of the society.

**Methods:**

The research group comprised Ist‐year physiotherapy and dentistry students from University School of Physical Education in Wroclaw and Wroclaw Medical University. The study consisted the following: psychological and health questionnaires, and stomatological examination with masseter muscles' electromyography.

**Results:**

In intraoral examination, symptoms of parafunctions were more frequently observed in physiotherapy students. The mean tone of masseters was higher also among physiotherapy students. The level of perceived stress was high in both groups, and the experienced borderline and incorrect results in Hospital Anxiety and Depression Scale were more frequently observed in dentistry students. The mean tone of masseter muscle was rising with the level of anxiety in physiotherapy group. Among dentistry, the tendency for rise of masseter muscle tone together with level of anxiety and depression was observed.

**Conclusions:**

Physiotherapy and dentistry beginners differ between each other's by prevalence of temporomandibular disorders (TMD) and oral parafunction symptoms. The level of perceived stress is high in both groups. The level of experienced anxiety and depression is higher in dentistry group. In both study groups, there is significant growth and tendency for simultaneous rise of masseter muscle tone accordingly to stress, anxiety, and depression indicators.

## Graphical abstract

As literature informs students' population is being put with greater level of stressful situations than the average representative of the society, scientific evidence exists to support the theory that psychoemotional factors play a significant role in the temporomandibular disorder (TMD) and oral parafunctions development. In our study, we have found the evidence to support those theses. The level of perceived stress is high in both groups (measured by PSS‐10). The level of experienced anxiety and depression is higher in dentistry group (evaluated by HADS). In both study groups, there exist a tendency for simultaneous growth of stress, anxiety, depression indicators, and masseter muscle tone.

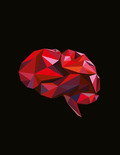

## 1 INTRODUCTION

Stress is defined as “a particular relationship between the person and the environment that is appraised by the person as taxing or exceeding his or her resources and endangering his or her well‐being” (Lazarus & Folkman, 1984). In the literature, students are described as a group of high‐risk stress exposure due to: high workload, high achievement pressure, good study performance expectations, high competitivity, limited free time, and limited sleep (Alzahem, Van der Molen, Alaujan, Schmidt, & Zamakhshary, 2011; Bathla, Singh, Kulhara, Chandna, & Aneja, 2015; Elani et al., 2014; Frączak et al., 2008; Jaworska, Morawska, Morga, & Szczepańska‐Gieracha, 2014; Morga, Podboraczyńska, Jaworska, & Szczepańska‐Gieracha, 2015; Park et al., 2012; Stallman, 2010). Numerous published studies state that stress can also negatively influence stomatognathic system by defusing and reducing psychoemotional overload on dental arches and surrounding tissues by teeth clenching or grinding (bruxing), cheeks and lips biting or sucking, pressing tongue against teeth as well as by performing many others occlusal and none occlusal parafunctions (Calixtre, Gruninger, Chaves, & Oliveira, 2014; Cioffi et al., 2017; Feu, Catharino, Quintão, & Almeida, 2013; Owczarek, Lion, & Radwan‐Oczko, 2020).

Oral habits or parafunctions are described as habitual functions that involve masticatory system and are not physiologically justified (Atsü, Güner, Palulu, Bulut, & Kürkçüoglu, 2019; Emodi‐Perlman et al., 2012). Oral habits are just like stress neither uncommon nor always harmful. However, when the parafunctional activity exceeds organs’ physiological adaptation ability, its function can be altered or even collapsed. Okeson and de Leeuw (2011) state the initial disturbance will appear in the tissue/organ with the lowest structure tolerance, in which stomatognathic system could be muscles, temporomandibular joint (TMJ) or teeth. Oral parafunctions are spread worldwide, and it is common for some people to have more than one. Numerous researchers have described positive association between stressful life events and the performance of multiple oral parafunctions (Emodi‐Perlman et al., 2012; Owczarek et al., 2020; Stocka, Kuc, Sierpinska, Golebiewska, & Wieczorek, 2015; Wieckiewicz et al., 2014). Across previous years, many studies contributed to the statement that oral parafunctional activity is associated with temporomandibular disorders (TMD) signs and symptoms—having an influence on its development (Ferreira, Simamoto‐Júnior, Novais, Tavares, & Fernandes‐Neto, 2014; Ohrbach et al., 2013; Panek et al., 2012; Więckiewicz, Paradowska‐Stolarz, & Więckiewicz, 2014).

Temporomandibular disorders, according to the WHO, is the third most common complaint reported in the dental offices with its prevalence ranging from 50% up to 80% in society (Stocka et al., 2015; Wieckiewicz et al., 2014). Its incidence peaks between being 20 and 40 years old (De Rossi, Greenberg, Liu, & Steinkeler, 2014). According to the current knowledge, the main causative and destructive role in the development of temporomandibular disorders are forces that significantly exceed adaptive capacity of stomatognathic system, deriving from increased tone of masticatory muscles in stressogenic situations and during parafunctional activities (Skármeta et al., 2019).

Stallman (2010) has proven that students are under higher stressful conditions than the rest of general society; hence, they are more vulnerable to develop TMD. Some researchers hypothesize that environmental academic stress contributes more toward TMD development in the students. Physiotherapy students are considered to have significant levels of perceived stress, anxiety, and depression during their academic years (Jaworska et al., 2014; Morga et al., 2015). In addition, some claim that dentistry is the most stressful medical profession (Bhat & Nyathi, 2019; Collin, Toon, O’Selmo, Reynolds, & Whitehead, 2019; Cooper, Watts, Baglioni, & Kelly, 2018).

The aim of the present study was threefold. The main goal was to compare psychoemotional state, electromyographical (EMG) masseter activity, and presence of signs and symptoms of oral parafunctional activity in two groups of junior medical students: physiotherapy and dentistry. These two groups of students were chosen because their education and clinical training are being held in different profile and scope universities, but with similar workload and clinical training. Secondly, we wanted to assess signs and symptoms of oral parafunctions prevalence in students’ oral cavity. Parafunctions are mainly performed under stress. Literature proves that stress‐induced tension of muscles is relieved by unconscious motoneuron activation of dental arches. It results in parafunction development and increased incidence of teeth contacts (Berger, Oleszek‐Listopad, Marczak, & Szymanska, 2015; Cioffi et al., 2017). Thirdly, we decided to explore the correlations between EMG results, signs and symptoms of oral parafunctions, oral manifestations of occlusal and nonocclusal parafunctions, and students’ psychoemotional condition.

## 2 MATERIALS AND METHODS

### 2.1 Participants

Total number of 105 volunteers took part in the research. The study consisted of two groups: 53 first‐year physiotherapy students from University School of Physical Education in Wroclaw and 52 dentistry beginners from Wroclaw Medical University. All of the participants gave their informed consent prior to their inclusion in the study. There were 35 women and 18 men in physiotherapy and 38 women and 14 men in dentistry group. Respectively, the mean age was 20.2 (*SD* ± 1.1) and 20.0 (*SD* ± 1.2) years old **(**Table 1).

**Table 1:** Characteristic of research participants

	Women	Men	Total	Mean age (±*SD*) [years]
Physiotherapy students	35	18	53	20.2 ± 1.1
Dentistry students	38	14	52	20.0 ± 1.2

The study design was prepared under guidelines for Good Clinical Practice from European Medicine Agency as well as The Declaration of Helsinki and was approved by Wroclaw Medical University Bioethical Committee (Nr.: 642/2017).

### 2.2 Procedure

The study was divided into two parts. First part consisted of medical and psychological questionnaires. In the second part, students underwent intraoral, clinical examination with bilateral, electromyographical evaluation of masseter muscles’ tone.

### 2.3 Instruments

In the medical questionnaire, participants were asked about their demographical data (sex and age) as well as about self‐reported temporomandibular joint area pain and self‐reported bruxism.

To obtain these information, we used modification of two first questions from TMD‐Pain Screener Form from DC/TMD Assessment Instruments (In the last 30 days did you have any pain or stiffness on awakening in your jaw or temple area on either side?) (Ohrbach, 2016) and the question: “Do you clench or grind teeth when asleep or awake? According to any information you may have”. Subsequently, students were assessed with two psychological instruments: Perceived Stress Scale (PSS‐10) by Cohen, Kamarck, and Mermelstein (1984) (Table 2) and Hospital Anxiety and Depression Scale (HADS) by Zigmond and Snaith (Zigmond & Snaith, 1983) (Table 3) to investigate and compare levels of perceived stress as well as depressive and anxiety symptoms among both groups. The study was conducted in May/June, one month before the examinations at the end of first academic year.

**Table 2:** Division of the groups according to obtained points in Perceived Stress Scale 10 (PSS‐10)

PSS‐10 points	Level of intensity of perceived stress
0–13	Low
14–19	Medium
20–40	High

**Table 3:** Division of the groups according to obtained points in Hospital Anxiety and Depression Scale (HADS)

HADS‐A or HADS‐D points	Level of experienced anxiety and depression
0–7	Correct
8–10	Borderline
11–21	Incorrect

*Perceived Stress Scale (PSS)* (Cohen et al., 1984) is a widely used questionnaire to assess the level of psychological stress–—subjective stressfulness of experienced situations and stress coping methods. PSS consists of 10 items (each scored from 0 to 4) and total score ranging from 1 to 40. It has good psychometric values with Chronbach's alpha internal consistency of 0.86 (Juczyński & Ogińska‐ Bulik, 2007). Previous research showed that PSS‐10 level can predict an increased risk for disease and levels of biological stress markers (Cohen et al., 1984; Juczyński & Ogińska‐ Bulik, 2007). Table 2 presents the way total points are assigned into three groups indicating: low, medium, or high level of perceived stress.

*Hospital Anxiety and Depression Scale (HADS)* (Zigmond & Snaith, 1983) was used to investigate the level of experienced anxiety and depression among participants. The Polish version (de Walden‐ Gałuszko & Majkowicz, 2000) consists of 14 items. Seven of them relate to anxiety (HADS‐A) and other seven to depression (HADS‐D). Score range varies from 0 to 21 for both HADS‐A and HADS‐D as single items score from 0 to 3. The instrument presents good psychometrical properties. Internal consistency measured with Chronbach's alpha was 0.9 for depressive subscale and 0.8 for anxiety subscale (Wichowicz & Wieczorek, 2011). Table 3 presents possible division into three groups (correct, borderline, and incorrect) based on obtained total scores (Bjelland, Dahl, Haug, & Neckelmann, 2002; de Walden‐ Gałuszko & Majkowicz, 2000).

### 2.4 Clinical evaluation

In clinical part of intraoral examination, the trained researcher evaluated the presence of occlusal parafunctions signs in oral cavity such as: gingival recessions, tooth wear symptoms in the occlusal surface, linea alba, and tongue crenations. In the present study, Miller's Gingival Recession Classification was used to evaluate gingival recession. At least one diagnosed gingival recession determined that student had this condition. Tooth wear was assessed according to the Tooth Wear Index (TWI) (Smith and Knight 1984). A lack of tooth wear was considered only when the subject obtained a total of 0 TWI score. TWI scores above 0 classified participants as having tooth wear. Subjects were also categorized according to the presence or absence of uni‐ or bilateral linea alba as well as tongue indentation.

**Figure 1:**
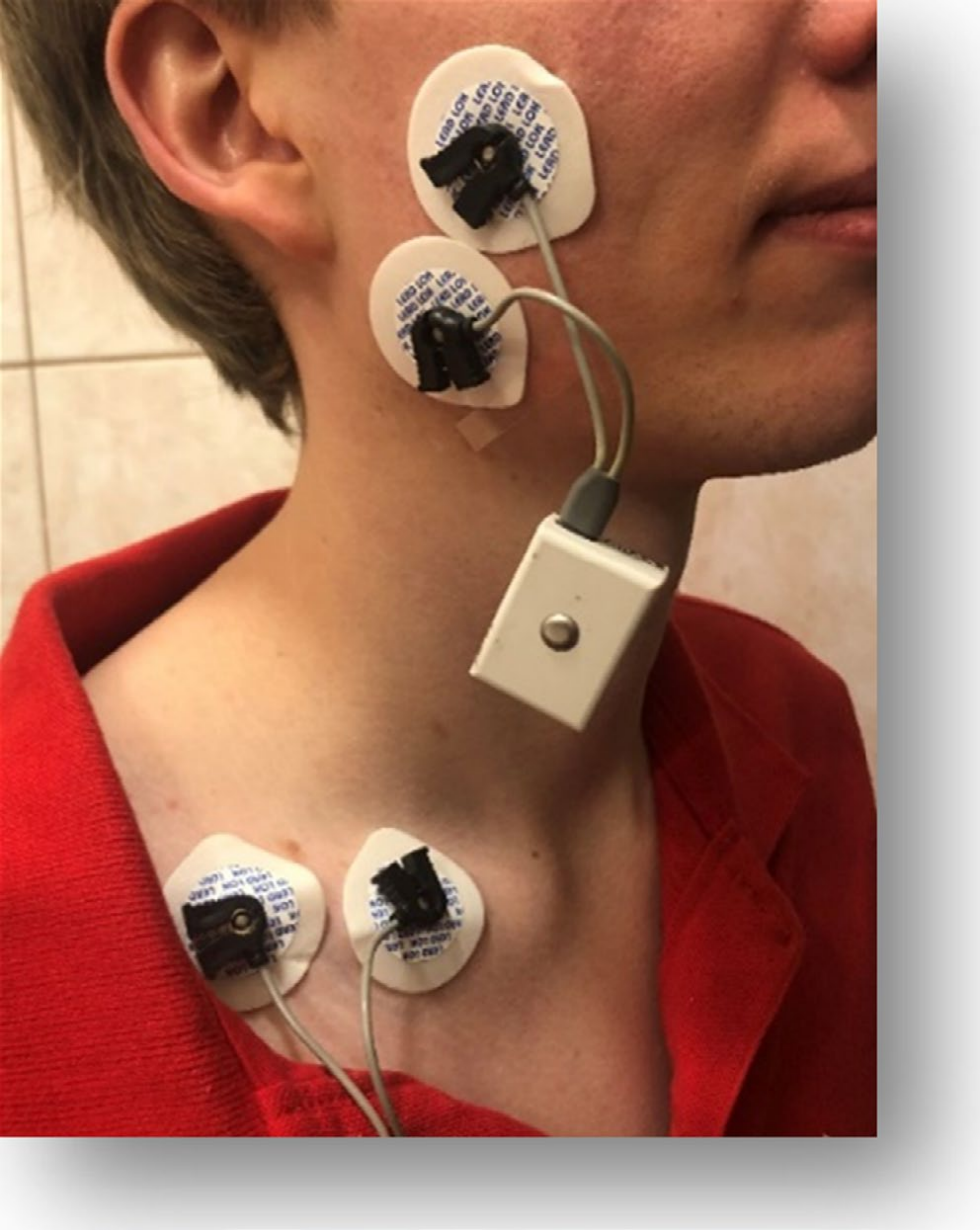
Electrodes placement during the electromyographical procedure

At the end of clinical examination, the EMG evaluation of left and right masseter in resting position of mandible was performed. Noraxon Clinical DTS device was used. Two disposable, silver chloride (Ag/AgCl) bipolar electrodes were placed on the skin overlying the center and in the line with masseters' muscle fibers. The zero electrode was attached in the area of supraclavicular pit (Figure 1). To reduce electrical impedance and to assure the best electrode fixation, the skin surface for the electrode was cleaned with 70% isopropyl alcohol. Before these procedures, all participants were individually informed about the routine of the EMG examination. Procedure was performed in a peaceful atmosphere, in closed, air‐conditioned (22°C) clinical room. Volunteers were asked to sit still in an upright position with hands laying on the tights. EMG muscle activity was measured for 15 s for each side. Measurements were always started from right masseter.

### 2.5 Statistical analysis

The collected data underwent statistical analysis. Verification of the equality of means of individual trials was performed by ANOVA variance analysis, or for non‐homogeneous variance groups, the verification was performed with the nonparametric test of the *U* Mann–Whitney (homogeneity of variance was checked by the Bartlett test). For discrete parameters, the incidence of features in the groups was analyzed by the Chi‐square test with Yates' correction. For some parameter, pairs correlation analysis was performed by calculating correlation coefficient *r*. *p*‐value of *p* ≤ .05 was considered statistically significant, whereas .05 < *p* < .1 was assumed to be likely to exist. Statistical analysis was performed by using computer packages of statistical programs EPIINFO Ver. 3.5.3.

## 3 RESULTS

### 3.1 Results from clinical questionnaire and examination

In the clinical questionnaire, 26% of physiotherapy students declared the presence of pain in TMJ area, but only 4 (8%) dentistry students. Self‐reported bruxism was reported by 19% of physiotherapy students and 13% of dental undergraduates.

In intraoral, clinical examination gingival recessions were observed in 6% of physiotherapy and 29% of dentistry students, while tooth wear symptoms in occlusal surface were detected among 85% of physiotherapy and only 21% of dentistry subjects. Linea alba was observed in 72% and 52% students, respectively. Tongue crenations, also called tongue indentations, were discovered in 53% of physiotherapy and 31% of dentistry students. Statistically important differences between research groups were detected in almost all of the cases (excluding self‐reported bruxism)—presented in Table 4.

**Table 4:** Medical questionnaire and intraoral examination results’ distribution

	Physiotherapy students *n* = 53	Dentistry students *n* = 52	*p* (Chi‐square test)
Pain in temporomandibular joint area	14 26%	4 8%	.011
Self‐reported bruxism	10 19%	7 13%	ns
Gingival recessions	3 6%	15 29%	.002
Tooth wear	45 85%	11 21%	.000
Linea alba	38 72%	27 52%	.037
Tongue indentations	28 53%	16 31%	.022

The EMG‐measured median values of masseters' tone in physiotherapy students were as follows: 66.4 µV (64.4 ÷ 68.5 µV) for right muscle (RM) and 67.9 µV (65.5 ÷ 70.0 µV) for left (LM). In dentistry students, these values for both of the muscles were as follows: 64.8 µV (RM 63.1 ÷ 66.6; LM 63.4 ÷ 66.7 µV) (Table 5).

**Table 5:** Electromyographical values of masseter muscle's tone

	Physiotherapy students *M* (25Q ÷ 75Q)	Dentistry students *M* (25Q ÷ 75Q)	*p* (Mann‐Whitney *U* test)
Tone of RM [µV]	66.4 (64.4 ÷ 68.5)	64.8 (63.1 ÷ 66.6)	.009
Tone of LM [µV]	67.9 (65.5 ÷ 70.0)	64.8 (63.4 ÷ 66.7)	.000

Abbreviations: LM, left masseter; RM, right masseter.

### 3.2 Results from psychological questionnaires PSS‐10 and HADS

The mean results in PSS‐10 indicated a high level of perceived stress in both groups—22 points (20.0 ÷ 25.0) among physiotherapy students and 21 (16.0 ÷ 24.0) among dentistry students (*p* = .088). The mean level of anxiety symptoms (HADS‐A) among dentistry students was between the border and high level (10; (*SD* ± 3.7)). Whereas for physiotherapy students, it was at the border level (8.11; (*SD* ± 3.1)). Although both groups presented increased level of anxiety, the result was statistically higher among dentistry students (*p* < .006). Although the results in depressive symptoms, HADS‐D can be considered as normal in both groups of participants (6 (*SD* ± 3.0) for dentistry students and 4 (*SD* ± 2.8) for physiotherapy students), dentistry students presented statistically higher levels (*p* < .002). The detailed results are described in Tables 6 and 7.

**Table 6:** Results of psychological test PSS‐10

	Physiotherapy students (*n* = 53)	Dentistry students (*n* = 52)	*p*
Median result of PSS‐10	22.0 (20.0 ÷ 25.0)	21.0 (16.0 ÷ 24.0)	.088^a^
Nr of students with low PSS‐10 results	1 2%	8 15%	.016^b^
Nr of students with medium PSS‐10 results	12 23%	15 29%	
Nr of students with high PSS‐10 results	40 75%	29 56%	

^a^ Mann‐Whitney *U* test

^b^ Chi‐square test.

**Table 7:** Results of psychological test HADS

	Physiotherapy students	Dentistry students	*p*
HADS mean value of A (anxiety) component	8 ± 3.1	10 ± 3.7	.006^a^
Nr of students with correct HADS‐A component results	27 51%	14 27%	.039^b^
Nr of students with borderline HADS‐A component results	14 26%	19 36.5%	
Nr of students with incorrect HADS‐A component results	12 23%	19 36.5%	
HADS mean value of D (depression) component	4 ± 2.8	6 ± 3.0	.002^a^
Nr of students with correct HADS‐D component results	46 86.8%	39 75%	ns^b^
Nr of students with borderline HADS D component results	5 9%	8 15%	
Nr of students with incorrect HADS‐D component results	2 4%	5 10%	

Abbreviation: HADS, Hospital Anxiety and Depression Scale.

^a^ ANOVA test

^b^ Chi‐square test.

### 3.3 Tone of masseter muscle and psychological symptoms

The PSS‐10 and HADS are psychological tools to define subjects’ level of perceived stress, anxiety, and depression exposure (Cohen et al., 1984; Zigmond & Snaith, 1983). Obtained points in each test allow to divide students into adequate subgroups.

In physiotherapy group, statistical analysis confirmed existing important differences between mean tone of left masseter in correct subgroup of HADS‐A and borderline subgroup as well as between borderline and incorrect subgroup.

Among dentistry participants, the tendency for statistical difference between mean value of electrical potential of only left masseter in correct and borderline subgroups of HADS‐A and HADS‐D was detected.

Although, there were no statistical differences between tones of masseters in different subgroups, but in many cases, values of masseters’ electrical potentials are considerably lower in low PSS‐10 and correct HADS subgroups then in other subgroups (Table 8).

**Table 8:** The assessment of the masseter muscle activity in the PSS‐10 and HADS‐based subgroups [µV]

**Physiotherapy**			
	**PSS‐10, low** Median (25Q ÷ 75Q) [µV]	**PSS‐10, medium** Median (25Q ÷ 75Q) [µV]	**PSS‐10, high** Median (25Q ÷ 75Q) [µV]
Masseter right	61.5 (only 1 person)	66.9 (63.4 ÷ 69.5)	65.4 (62.7 ÷ 68.5)
Masseter left	62.0 (only 1 person)	66.3 (64.7 ÷ 70.4)	67.7 (64.6 ÷ 70.5)
	**HADS‐A, correct** Median (25Q ÷ 75Q) [µV]	**HADS‐A, borderline** Median (25Q ÷ 75Q) [µV]	**HADS‐A, incorrect** Median (25Q ÷ 75Q) [µV]
Masseter right	66.1 (62.9 ÷ 69.1)	65.6 (64.0 ÷ 68.8)	67.0 (64.5 ÷ 69.4)
Masseter left	66.0(63.9 ÷ 68.3)^a^	67.6 (66.6 ÷ 71.6)^a^, ^b^	70.0 (64.4 ÷ 69.6)^b^
	**HADS‐D, correct** Median (25Q ÷ 75Q) [µV]	**HADS‐D, borderline** Median (25Q ÷ 75Q) [µV]	**HADS‐D, incorrect** Median (25Q ÷ 75Q) [µV]
Masseter right	62.9 (61.3 ÷ 63.1)	67.0 (64.2 ÷ 69.1)	62.6 I 71.5 (only 2 person)
Masseter left	65.3 (65.1 ÷ 70.3)	67.1 (64.7 ÷ 70.5)	69.2 I 88.3 (only 2 person)
**Dentistry**			
	**PSS‐10, low** Median (25Q ÷ 75Q) [µV]	**PSS‐10, medium** Median (25Q ÷ 75Q) [µV]	**PSS‐10, high** Median (25Q ÷ 75Q) [µV]
Masseter right	62.8 (61.7 ÷ 67.9)	65.7 (64.2 ÷ 67.8)	66.5 (63.9 ÷ 67.8)
Masseter left	65.6 (64.7 ÷ 66.8)	67.1 (65.2 ÷ 68.3)	66.8 (63.9 ÷ 67.8)
	**HADS‐A, correct** Median (25Q ÷ 75Q) [µV]	**HADS‐A, borderline** Median (25Q ÷ 75Q) [µV]	**HADS‐A, incorrect** Median (25Q ÷ 75Q) [µV]
Masseter right	65.3 (64.1 ÷ 67.4)	65.7 (63.2 ÷ 68.3)	66.3 (63.6 ÷ 68.3)
Masseter left	63.3 (64.7 ÷ 70.5)^c^	67.0 (65.6 ÷ 68.2)^c^	67.0 (64.5 ÷ 68.1)
	**HADS‐D, correct** Median (25Q ÷ 75Q) [µV]	**HADS‐D, borderline** Median (25Q ÷ 75Q) [µV]	**HADS‐D, incorrect** Median (25Q ÷ 75Q) [µV]
Masseter right	66.1 (63.6 ÷ 67.9)	65.9 (63.4 ÷ 68.3)	64.7 (63.9 ÷ 65.4)
Masseter left	66.6 (64.5 ÷ 67.5)^c^	68.1 (66.8 ÷ 69.6)^c^	66.9 (65.0 ÷ 67.8)

^a^ Statistical difference between correct HADS and borderline HADS.

^b^ Statistical difference between borderline HADS and incorrect HADS.

^c^ Tendency for statistical difference between correct HADS and borderline HADS. Mann–Whitney *U* test.

Moreover, performed statistical analysis revealed that positive correlation exists between EMG‐measured tone of masseter muscle and obtained points in psychological test PSS‐10 in male physiotherapy group (*r* = .54, *p* < .022), but only for left masseter. Correlation was not observed for right masseter, and also for left masseter when all physiotherapy subjects were considered as a one group. [not included in a table].

Additionally, positive correlation was observed in male physiotherapy students between tone of right masseter and results from HADS test‐D component (*r* = .48, *p* < .042). [not included in a table].

In all other cases, which are between masseters muscles’ tones of dentistry students and psychological components, between physiotherapy beginners and HADS‐A test scores, statistical analysis of variables did not find any statistically important correlations. [not included in a table].

### 3.4 Tone of masseter muscle and oral manifestation of parafunctions and self‐reported symptoms in stomatognathic system

Statistical analysis of correlations between obtained EMG values of right and left masseters’ tone and oral mucosa manifestations of parafunctions and self‐reported symptoms in stomatognathic system such as: gingival recessions, tooth wear symptoms, linea alba, tongue crenations, self‐reported bruxism, and pain in TMJ area revealed only one existing correlation. This sole correlation was between tone of left masseter and tongue indentations in both physiotherapy (*p* < .000, *r* = .17) and dentistry (*p* < .048, *r* = .23) group. The other research variables did not show the tendency for correlation. [not included in a table].

### 3.5 Psychological burden and oral manifestation of parafunctions and self‐reported symptoms in stomatognathic system

In the same manner as above correlations between emotional condition of participants (evaluated by psychological tests PSS‐10 and HADS) and their intraoral parafunction manifestations, self‐reported bruxism and pain in TMJ area were searched.

In physiotherapy subjects, this analysis showed the positive correlation between PSS‐10 test results and the presence of linea alba (*r* = .29, *p* < .033) and the tendency for the positive correlation between HADS‐A test scores and self‐reported pain in TMJ area (*r* = .23, *p* < .093). Statistical analysis did not find correlation between intraoral variables and results of HADS component D test.

Among dentistry students, the only positive correlation that was found was the correlation between HADS‐A score and the presence of linea alba in the examined subjects (*r* = .34, *p* < .019).

When other variables (both psychological and clinical questionnaire and examination results) in both study groups were taken into consideration, the analysis did not return statistically important concatenation. [not included in a table].

## 4 DISCUSSION

The study confirmed the hypotheses about the increased level of perceived psychoemotional burden among physiotherapy and dentistry students. The vast majority of all students were considered to perceive medium and high levels of stress based on PSS‐10 results. The percentage of subjects with high values in the PSS‐10 was 75% for the physiotherapy juniors and 31% for dentistry students. Low level of perceived stress was represented by only one physiotherapy undergraduate. The average scores obtained by all students placed them in a group with a high level of perceived stress. These high values indicated a strong psychoemotional overload that might alter normal psychophysical functioning which may lead to systemic health problems.

The results are consistent with the meta‐analysis of stress research in the dental students community performed by Elani et al. (2014). Their results showed that participants of 34% of all analyzed studies (on stress levels among dentistry students) presented high values of experienced stress and 54% medium stress levels.

Until now, stress levels among physiotherapy students have not been as widely studied as among dentistry students. Among 5th‐year physiotherapy students of Wrocław University of Physical Education, Morga et al. (2015) obtained opposite results to ones presented in our work. Only 7% of her studied group obtained high levels of perceived stress while 63% presented low levels. Those differences in the obtained values of psychological tools could be explained with the differences between study groups. Morga et al. (2015) study participants were at the final stage of education which could result with greater emotional relaxation associated with the anticipated completion of the education process.

Other researchers also obtained lower levels of perceived emotional overload among physiotherapy students than presented in this work. Sabih, Siddiqui, and Baber (2013) study of 1st‐ and 2nd‐year students presented 46% of subjects with increased rates of perceived stress. Students from three countries, Israel, Sweden, and Australia, described in a comparative study by Jacob et al. (2012), declared the average PSS‐10 results indicating medium stress exposure.

Literature analysis shows that not only increased levels of stress are common in the student population. Studies show an increased percentage of students with anxiety and depressive disorders. Additionally, it was proved that TMD positively correlates with the occurrence of the depression and anxiety. Researchers emphasize that the complication of temporomandibular disorders by anxiety and depression extends its duration of treatment and worsens the prognosis. It is also associated with a higher percentage of people with pain located in the temporomandibular joint (Ivkoviv, Mladenovic, Petkovic, & Stojic, 2008; Koralewski, Koczorowski, & Gracz, 2001; Stocka, Sierpinska, Kuc, & Golebiewska, 2017; Yachida et al., 2012).

Ali, Fatima, Ilyas, Khan, and Abbassi (2016) reported anxiety and depressive symptoms in 68% of students starting dental studies, and 72% among fifth‐year students. In turn, Galan, Rios‐Santos, Polo, Rios‐Carrasco, and Bullon (2014), in the assessment of Spanish dentistry students, found depressive symptoms in 12% of first‐year students and in 4% of fifth‐year representatives, the difference between years was statistically significant. In Jaworska et al. (2014) study among the population of future physiotherapists of the Wrocław University of Physical Education, the authors observed serious symptoms of depression in 8% of respondents. In an earlier study by Szczepańska, Klin, Jaroszewska, and Ciesielski (2008), also among students of physiotherapy at the University of Physical Education in Wrocław, the percentage of people with severe depressive symptoms was lower—5%. However, the differences may be explained by different workload, learning new coping mechanisms during the 5‐year long course as well as using different methodology, especially different assessing scales.

The results obtained in this work indicated increased levels of anxiety symptoms among the studied population. The average values obtained in the HADS‐A test (anxiety) by student groups placed them in the border group characterized by mild anxiety symptoms. However, nearly ¼ of future physiotherapists and more than 1/3 of first‐year dental adepts were in the range of people with incorrect HADS‐A test values, indicating the presence of pathological anxiety disorders. Jadoon, Yaqoob, Raza, Shehzad, and Zeshan (2010), by using the same psychological tool, also described a high percentage (45%) of people with increased values for anxiety and depression in the population of first‐ and last‐year medical students.

The analysis of the own results of the HADS‐D subtest showed that both student groups obtained average values—indicating no depressive disorders. However, the average number of points in HADS‐D in the group of first‐year dentistry students was significantly higher than in the physiotherapy group. The mean value was approaching the limit, which makes it possible to diagnose mild depressive disorder. It can be seen that there were more people within dentistry student group showing mild (15%) and severe (10%) depressive symptoms than in the physiotherapy adepts. It may be associated with more frequent physical activity of physiotherapists, resulting from the study program, compared to dentistry students. Many authors emphasize that regular physical activity is a mental health protective factor, and depression rates are lower in among professional athletes than in the general population (Jaworska et al., 2014; Morga et al., 2015; Żaroń & Piskunowicz, 2017).

In own study, statistically significantly more first‐year students of the University of Physical Education (26%) reported complaints in the temporomandibular joint than dentistry students—8%. Więckiewicz et al. (2014) and Panek et al. (2007) declared higher prevalence of self‐reported symptoms within the temporomandibular joint among dental students, respectively, 47%, 38%, and 57%. This difference can be explained by the greater knowledge and self‐awareness of 5th‐year students described in those papers (Panek et al., 2007; Wieckiewicz et al., 2014).

Observation of the occurrence of symptoms such as: tooth wear (TW), linea alba, tongue indentations, and gingival recessions may indicate the performance of parafunctional activities like teeth clenching, bruxing, or buccal mucosa chewing (Ferreira et al., 2014; Feu et al., 2013; Owczarek et al., 2020). In the conducted study, TW was the most common symptom of parafunctions among physiotherapists. On the other hand, linea alba was the most common sign of parafunction observed in dental group (52%). Tongue indentations were found in slightly lower percentage, 53% and 31%, respectively. And gingival recessions in 6% of physiotherapy students and 29% of first‐year dentistry students. The differences between the groups were statistically significant.

In our study, linea alba was the most frequently observed symptom of parafunction (in the total group)—presented by 72% of physiotherapists and 52% of dental students. Similarly, Anisuzzaman and Rungsiyanont had a high percentage of linea alba, 39.6% and 42%, respectively (Anisuzzaman, Khan, Hasan, Adnan, & Afrin, 2019; Sorasun, Photipakde, Piboonratanakij, Patrayu, & Kajorndej, 2012).

There was also a tendency to observe linea alba more frequently in connection with the increase of anxiety symptoms (HADS‐A). In the own study, linea alba was the only observed change which showed a tendency to occur more frequently in the case of diagnosed anxiety disorders.

Population studies on the frequency of gingival recession showed a varied level of its prevalence, which was 19% in Swedes, 30% in the population of Poles, and 50% in Germany (Cortellini & Bissada, 2018; Dominiak & Gedrange, 2014; Sulewska et al., 2017). In the studies of Kozłowski, Konopka, Karolewska, Mendak, and Szulc (2003) and Bochniak, Tyrzyk, and Kryspin (2003)) the presence of gingival recession was found in 35% of the examined students of the first and last year of dentistry. These results were higher than those obtained in our study (29%).

An increased percentage of gingival recessions among dental students was also observed in many other studies (Coral, Sabogal, & Serrano, 2014; Mythri et al., 2015), in which statistical analyzes showed positive correlations between intensified hygiene procedures, greater dental knowledge, and the number of current gingival recessions.

The results of studies on the presence of TW in the general population indicate a large discrepancy, which ranges from 13% to 98% (Suchetha, Sravani, Mundinamane, & Chandran, 2014). In our study group (105), TW was observed in 53% of total respondents. In individual study groups, this percentage was 85% among physiotherapists and 21% among dental students. The differences between the groups were statistically significant. The results of a study carried out in groups of young adults (aged 19–22 years) also showed a high percentage of TW in the studied population (at the level of 77%) (Bartlett, Harding, Sherriff, Shirodaria, & Whelton, 2011). On the other hand, in the study of Žuvela, Alajbeg, Illeš, and Tarle (2011) among young military sailors, the spread of TW was diagnosed at the level of 22%.

Many authors hypothesize, contrary to the common opinion, that bruxism is not always correlated with an increased incidence of TW. Hirsch, John, Lobbezoo, Setz, and Schaller (2004), in the studied group of teenagers, did not find statistically significant correlations between the prevalence of TW and the declared ailments in the area of TMJ. Similarly, Pergamalian and Žuvela did not show a correlation between diagnosed bruxism and the presence of TW (Pergamalian, Rudy, Zaki, & Greco, 2003; Žuvela et al., 2011). The above reports can help to understand own results as our statistical analysis did not show significant correlations. In the group of physiotherapists, observing the highest percentage of prevalence of TW, there was no positive correlation between the declared grinding and clenching of teeth and pain, which in this group was at a low level. It should be noted that the reports from the literature presented above are very divergent. This may also result from different methods of TW assessment.

The presence of tongue indentation seems to be widespread in the general population. In the Emodi‐Perlman study, they occurred in 4%, in the Ajalbeg study in 41% (Alajbeg, Zuvela, & Tarle, 2012; Emodi‐Perlman et al., 2012). In turn, Umoh described their presence in up to 58.5% (Umoh & Azodo, 2013), and Wetselaar as much as 80% in their studied groups (Wetselaar, Vermaire, Visscher, Lobbezoo, & Schuller, 2016). In our study, this percentage was 53% for physiotherapists, and 31% for first‐year dentistry students.

Frequent prevalence of TW, linea alba, and tongue indentation in combination with a low percentage of TMJ area complaints, bruxing and gingival recessions, among the population of physiotherapists, may indicate the presence of other determinants involved, but not included in the study. Perhaps not without significance for the results obtained is the fact that these students have more frequent and intense physical activities. Equally frequent results of the symptoms of TMD in the group of students of dentistry and physiotherapy were presented by Koralewski et al. (2001), who emphasized that a certain correlation can be observed between some psychoemotional features and the condition of the stomatognathic system in physiotherapy students.

Increased psychoemotional load and parafunctional activities within stomatognathic system may lead to the development of functional dysfunctions. The results of many studies prove that stress causes an increase in the muscular activity of the stomatognathic system, which in turn increases and prolongs the duration of dental contacts, which can lead to tissue destruction, especially in young individuals (Cioffi et al., 2017; Kindler et al., 2012; Stocka et al., 2015).

The median tone of masseter muscles in first‐year dentistry students for both, left and right, masseter muscles was identical and amounted to 64.8 µV. In contrast, the values of the average right and left masseters’ tone among physiotherapy students were higher and amounted to 66.4 and 67.9 µV, respectively. Differences between tensions between student groups were statistically significant only for LM. Komiyama et al. (2008) and Manfredini, Fabbri, Peretta, Nardini, and Lobbezoo (2011) studies investigated relationships between masticatory muscle activity using electromyography and anxiety. They reported that an above median value for anxiety and depression was positively correlated with EMG‐assessed non‐functional nocturnal masticatory activity. Ivkoviv et al. (2008) and Yachida et al. (2012) used EMG to find correlations between muscle activity and depression. Ivkovich et al proved lower incidence of TMD and chronic muscle pain in patients using antidepressants. Yachida et al. found a positive correlation between increased activity of face muscles and depression. Moreover, Stocka et al. (2017) detected the increase in the mean tone of masseter muscles in maximal voluntary clench in the group with depressive disorders among young adults. Own findings harmonize with the discussed results. Obtained mean values of RM and LM tone were rising in both, physiotherapy and dentistry groups, when psychological tests’ results were taken into account. There was a significant difference in mean tone of LM in physiotherapy adepts in HADS‐A scores‐defined subgroups. Additionally, among dentistry students in anxiety (HADS‐A) and depression (HADS‐D) subgroups, the tendency for statistically important difference for the mean tone of LM was observed. Research should be continued to confirm obtained results.

## 5 CONCLUSIONS

1. Physiotherapy and dentistry students differ between each other in prevalence of oral parafunction symptoms.
2. The level of perceived stress is high in both groups (measured by PSS‐10). The level of experienced anxiety and depression is higher in dentistry group (evaluated by HADS).
3. In both study groups, a tendency for simultaneous growth of stress, anxiety, depression indicators, and masseter muscle tone exists.
4. There is a need for more research in this area.

## CONFLICT OF INTEREST

The authors declare that there is no conflict of interest regarding the publication of this paper.

## AUTHOR CONTRIBUTION

Joanna Elżbieta Owczarek and Małgorzata Radwan‐Oczko involved in study concept and design. Joanna Elżbieta Owczarek and Katarzyna Lion analyzed and interpreted the data. Joanna Elżbieta Owczarek drafted the manuscript. Małgorzata Radwan‐Oczko, Joanna Elżbieta Owczarek, and Katarzyna Lion critically revised the manuscript for important intellectual content. Joanna Elżbieta Owczarek statistically analyzed the study. Małgorzata Radwan‐Oczko supervised the study.

### Peer Review

The peer review history for this article is available at https://publons.com/publon/10.1002/brb3.1797.

## Data Availability

The data that support the findings of this study are available from the corresponding author upon reasonable request.
